# Indian Expert Consensus on Hybrid Cardiac Rehabilitation in the Management of Cardiovascular Disease

**DOI:** 10.7759/cureus.107135

**Published:** 2026-04-16

**Authors:** Jay Shah, Girish B Navasundi, Soumitra Kumar, Sreenivas Kumar, Chetan Gharat

**Affiliations:** 1 Cardiology, Ananta Hospital, Ahmedabad, IND; 2 Interventional Cardiology, Apollo Hospitals, Bannerghatta, Bengaluru, IND; 3 Cardiology, Fortis Hospital, Anandpur, Kolkata, IND; 4 Cardiology, Apollo Health City, Hyderabad, IND; 5 Medical Affairs, Lupin Digital Health, Mumbai, IND

**Keywords:** adherence, cardiac experts, digital telemedicine, level of evidence, quality of life

## Abstract

Cardiac rehabilitation (CR) is a structured, multidisciplinary, supervised program designed for patients recovering from cardiovascular (CV) events. This consensus aims to establish expert recommendations regarding hybrid CR (HCR) in the management of cardiovascular disease (CVD) in India. The objective was to review the existing literature and develop Indian expert opinions on HCR for CVD management. A comprehensive review of the current literature on HCR was conducted by experts. Based on the evidence gathered, the experts confirmed 10 statements regarding the management of CVD using HCR. These statements were presented at a conference booth, during a conference, where responses were collected from cardiac experts. The statements were graded based on the level of evidence: Level A evidence, indicating strong evidence from randomized controlled trials (RCTs) or meta-analyses; Level B, moderate evidence from RCTs with surrogate measures, observational studies, or meta-analyses; and Level C, weak evidence from observational studies, surrogate measures, or expert opinion. Of these 10 statements, six received Level A evidence, two received Level B evidence, and the remaining two received Level C. Feedback was documented, and the statements were finalized into a consensus. The consensus emphasizes the importance of acceptance, duration, monitoring, medication adherence, and the structure of HCR, as well as the roles of the CR team and the outcomes of HCR for patients with CVD. HCR is reported to enhance patients' adherence to medication, exercise, and diet, improve quality of life, and reduce the risk of CVD. Thus, the study successfully developed an expert consensus on HCR, offering valuable insights for its future integration into CR practices in India.

## Introduction and background

Cardiac rehabilitation (CR) is a structured, supervised program designed for patients recovering from cardiovascular (CV) events, including heart failure (HF), congenital heart disease, recent myocardial infarction (MI), and major cardiac procedures such as angioplasty, bypass surgery, valve surgery, and transplantation [1]. It combines exercise training, patient education, and social support to reduce the risk of future cardiovascular disease (CVD) [2].

Despite strong evidence, CR remains underutilized [3]. Several barriers contribute to this gap, including limited healthcare resources, poor access, lower educational levels, and high costs [4]. To overcome these challenges, alternative models of CR delivery have been developed to improve accessibility and cost-effectiveness. One such approach is hybrid CR (HCR), which combines supervised center-based sessions with home-based care [3,5]. This model offers greater flexibility and clinical rigor [6], thereby improving patient adherence and reducing delivery costs [7], further improving access and outcomes for patients with coronary artery disease (CAD), HF, and low-risk acute coronary syndrome (ACS) [6-8]. However, robust clinical evidence on HCR implementation, outcomes, and feasibility in the Indian context is limited.

Typically, the HCR programs begin with 2-11 weeks of center-based sessions, followed by 10-22 weeks of home-based sessions. The program includes three to five sessions per week, focusing on walking or cycling for 25-35 minutes at 60%-75% of the patient's maximal heart rate [7]. The successful implementation of HCR typically involves a multidisciplinary team, supported by digital health tools such as smartphone applications and a clinician dashboard accessible by healthcare professionals and patients. An equitable onboarding process for patients is essential to ensure access for individuals with varying levels of digital literacy. Gathering user feedback, implementing improvements based on it, and evaluating clinical efficacy are also critical steps in the process [5].

The HCR has proven effective as a hospital-based program in reducing pain components and lowering costs compared to traditional hospital-based delivery [9]. It also improves patient adherence to CR [10]. This can enhance the quality of life (QoL) and reduce CV risk factors in patients with HF, making it a viable option for both urban and rural populations [11]. The HCR offers enhanced monitoring during the initial exercise phase, supporting patients who require direct professional supervision for safe training by healthcare professionals [6].

Studies have reported that HCR can be an effective option for patients with CVD. However, to our knowledge, there is no expert opinion or consensus from India on this subject, followed by limited evidence. To address this gap, a group of Indian cardiac experts has come forward to review the existing literature and develop recommendations to guide Indian physicians in utilizing HCR to improve outcomes for patients with CVD.

## Review

Methods

Search Strategy and Evidence Summary

An expert in the field conducted a targeted literature review to identify key topics and current evidence related to HCR. Relevant studies were identified through database searches and manual screening of references. The evidence gathered was used to inform statement development. The key findings informed the drafting of 10 preliminary statements.

Consensus Process

These statements were presented in person at the conference booth. Participation was open to cardiac experts attending the conference, with a total of 162 experts responding. Participants rated each statement on a five-point scale as presented in Table 1 [12].

**Table 1:** Level of agreement

Level	Description
I	Accept completely
II	Accept with some reservations
III	Accept with major reservations
IV	Reject with some reservations
V	Reject with major reservations

**Figure 1:**
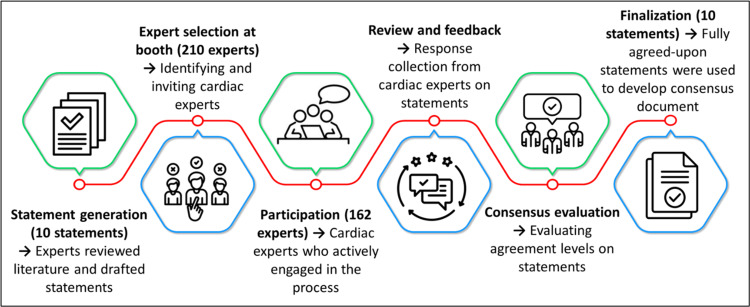
Consensus-building process Image credits: Created by the authors using Microsoft PowerPoint (Microsoft Corporation, Redmond, USA)

These responses were gathered to assess the participants' opinions and perspectives on the statements. Consensus on a statement was achieved when 80% or more of the participants selected either “accept completely” or “accept with some reservation.” Conversely, a statement was refuted if 80% or more of the participants chose either “reject with major reservation” or “reject with some reservation” [12]. Voting results were then analyzed, and based on the feedback, a consensus document was developed. This document was subsequently reviewed and approved by the experts. The consensus-building process is illustrated in Figure 1.

Results

Acceptance

Statement: The HCR offers flexibility and convenience compared to in-center programs, potentially improving adherence rates.

Level of evidence: A; level of agreement: I - 83.33%, II - 16.67%.

The HCR has demonstrated improved adherence in patients with CAD compared to traditional CR. The Improving ATTENDance (iATTEND) trial reported similar attendance patterns between facility-based and home-based CR [13]. Hybrid models combining initial hospital training with tele-monitored home exercises have shown particularly high adherence in women recovering from MI [14]. Furthermore, HCR also supports both men and women post-MI, with men demonstrating higher rates of return to work following program completion [10]. Scientific statements from organizations such as the American Association of Cardiovascular and Pulmonary Rehabilitation, the American Heart Association, and the American College of Cardiology suggest that incorporating technological tools into home-based CR models can enhance accessibility and adherence rates when compared to center-based CR [15].

The grading of the statements was based on the level of evidence presented in Table 2 [16].

**Table 2:** Grading based on level of evidence RCT, randomized controlled trial

Code	Definition	Interpretation
A	RCT or meta-analysis of RCTs; the single trial is enough if sufficient power and without important limitations.	Strong evidence. Evidence of high certainty. It is unlikely that future studies will change the effect estimates substantially.
B	RCT with surrogate measures, observational studies, and meta-analysis, including the above study types.	Moderate evidence. Evidence from some future studies may modify, at least the magnitude of, the effect estimate.
C	Observational studies of surrogate measures; any study type may be downgraded to level C due to limitations and expert opinion.	Weak evidence. Evidence of low certainty. Future studies may change the effect estimate substantially.

Statement: The HCR provides patients with self-paced technology tutorial videos tailored to various levels of digital literacy for additional support.

Level of evidence: B; level of agreement: I - 80.86%, II - 17.28%, IV - 1.85%.

The HCR program provides self-paced tutorial videos tailored to technology, designed for various levels of digital literacy [5,17]. According to the American Heart Association, ensuring patient access to education and training on digital technology is essential to prevent worsening health disparities when implementing a digital health tool in CR [18]. In HCR phase II services (outpatient multidisciplinary program including exercise and non-exercise strategies), approximately 5% of patients were ineligible due to poor computer literacy [19], highlighting the importance of providing technology training or alternative support for patients with lower digital literacy.

Duration

Statement: A typical HCR program should last three months, with the first month in-center followed by two months of remote CR support.

Level of evidence: A; level of agreement: I - 86.42%, II - 12.96%, III - 0.62%.

The QUALIREHAB multicenter, randomized controlled trial (RCT) evaluated a 12-week HCR program, which combined center- and home-based components, multidisciplinary care, and physical activity sessions [20]. In another RCT, participants received 10 center-based supervised exercise sessions with counseling over four to six weeks, followed by home-based support through telephone calls and text messages during 8-12 weeks [21]. Similarly, a study, which was part of the HYCARET (Hybrid Cardiac Rehabilitation Trial), involved patients with CAD who followed a 12-week hybrid exercise-based CR program [3]. Additionally, a feasibility study of HCR incorporated a four-week introductory period with three supervised exercise sessions per week, followed by a nine-week hybrid phase consisting of two supervised sessions and one home-based session per week [22]. Overall, the structure of these HCR programs differs across studies, but generally begins with a center-based component lasting between 2 and 11 weeks, followed by a home-based component lasting 10-22 weeks [7].

Monitoring

Statement: Remote monitoring tools (e.g., wearable devices) should be an integral part of hybrid rehabilitation to track vitals and activity.

Level of evidence: A; level of agreement: I - 80.25%, II - 2.47%, III - 17.28%.

Outpatient rehabilitation services are often overburdened, creating a need for alternative solutions and modern technology integration. Telerehabilitation offers a practical approach, enabling functional assessment at home through wearable devices and modern algorithms. This model, aligned with point-of-care testing trends, enables patients to receive care at their location, addressing high demand for rehabilitation services [23]. Wearable health devices allow continuous tracking of vital signs during daily activities or clinical settings with minimal discomfort [24]. Smart wearable sensors can enhance patient care, support physician-patient relationships, increase patient autonomy, and enable remote monitoring, thus revolutionizing healthcare management and overall cost [25].

A meta-analysis reported that wearable physical activity monitors, combined with exercise prescription (a personalized plan that outlines the type, intensity, duration, and frequency of exercise), significantly enhance cardiorespiratory fitness during the maintenance phase of CR [26]. Similarly, in an RCT, exercise tolerance improved in patients with CAD enrolled in remote CR programs guided by exercise coaching and wearable devices. The long-term benefits are particularly enhanced with online coaching supported by real-time monitoring [27].

Additionally, a longitudinal observational study using an iPhone and Apple Watch (Apple Inc., California, USA) indicated that these devices accurately predict the in-clinic six-minute walk test (6MWT), demonstrating their potential to remotely monitor frailty and functional capacity (FC; ability to perform physical activities and daily tasks safely and effectively) in patients with CVD [28]. Real-world evidence also indicates that applications such as Lyfe (by Lupin Digital Health Pvt. Ltd., Mumbai, India), combined with wearable devices, improve CV health, support medication adherence, and facilitate lifestyle modifications in patients with CAD or post-percutaneous coronary intervention [29,30].

Medication Adherence

Statement: Digital reminders and telephonic follow-ups should be implemented in hybrid programs to ensure medication adherence.

Level of evidence: A; level of agreement: I - 100%.

Non-adherence can negatively impact health and hinder treatment success. In a study, a mobile app-based medication reminder system was introduced that helps patients, especially the elderly and illiterate, who struggle to read medication labels, which can lead to incorrect medication consumption. This study presented a cost-effective, mobile app-enabled portable system designed to remind patients to take medications at home. The system calls patients five minutes after an alarm if they have not opened their prescribed medication box. It also provides routine health checks for the elderly, making it suitable for individuals living independently or in households with busy family members [31].

In a single-center feasibility study, post-MI patients treated with percutaneous or surgical coronary revascularization were offered a physical activity monitor connected to a customized app during their initial rehabilitation assessment. The digitally monitored group showed a trend toward higher metabolic equivalent of the task at 12 weeks (p<0.059). Integrating technology with in-person CR demonstrated promising outcomes, allowing for personalized content delivery and potential expansion [32]. A two-arm parallel RCT including patients with coronary heart disease evaluated a 12-week technology-assisted program guided by the Health Belief Model. The intervention included three supervised center-based exercise sessions, a fitness watch, six educational videos, and weekly video calls. Participants completed 85.5% of the 60 scheduled exercise sessions. Post-intervention surveys indicated that 23.1% of participants were “very satisfied” and 76.9% were “satisfied” with the program. These findings suggest that the technology-assisted HCR is feasible and effective in enhancing exercise self-efficacy, exercise capacity, and health-promoting behaviors [33].

Another study compared in-person, hybrid, and virtual CR outcomes using the 6MWT. Patients in the hybrid and virtual groups received synchronous video exercises and/or asynchronous telephone visits. Results showed improvements in the 6MWT, BP control, and anxiety. Feedback highlighted the flexibility of virtual CR, the importance of patient-staff relationships, and the need for organizational support. Overall, hybrid and virtual CR provided similar benefits to in-person CR, enhancing accessibility without compromising outcomes [34]. Due to the reminders that encouraged medication adherence, 92.5% of patients with CAD or ACS, who used the Lyfe app, did not forget to take their prescriptions, according to a prospective real-world evidence study that evaluated the app’s efficacy [35].

Structure

Statement: A hybrid program should include in-center patient assessment, goal setting, in-center supervised sessions, followed by remote monitoring, virtual dietary counseling, and cardio therapy sessions to achieve desired outcomes.

Level of evidence: A; level of agreement: I - 97.53%, II - 2.47%.

A qualitative study exploring patients’ perceptions of virtual models of CR, either hybrid (combining center-based and virtual) or virtual-only, reported that patients’ preferences, technology use, and delivery of core components should be considered when planning virtual programs. Support for CR patients should be provided outside program participation, starting at referral and extending beyond program completion [36].

A multidisciplinary team implemented a 12-week HCR program combining in-center and virtual sessions through an evidence-based digital health platform. The program followed a five-phase framework from recruitment to graduation. Phase 1 streamlined patient identification using Epic reports and auto-referral order sets. Phase 2 established flexible onboarding, including instructional videos tailored to varying levels of digital literacy, and assembled a diverse CR team to engage traditionally underrepresented patients. Phase 3 introduced a structured weekly coaching curriculum to enhance patient engagement. Phase 4 refined risk criteria to account for the absence of exercise data during hospitalization and incorporated two in-person safety assessments. Phase 5 explored the patient’s interest in advocacy after graduation, potentially through social media support groups. Overall, the hybrid program offered a scalable solution for patients unable to attend traditional CR regularly [17].

A pilot RCT evaluated the TecHCR intervention, which combined three center-based supervised exercise sessions with home-based exercise self-monitoring using a fitness tracker. Participant data was shared through a web-based application for remote monitoring. Participants also received six audiovisual educational videos via a messaging app and weekly follow-up via video or telephone calls. The results suggest that TecHCR is a feasible alternative for promoting health behaviors in patients with coronary heart disease [33].

Statement: A hybrid program should include a diet based on primary medical conditions and co-morbidities, and tailored according to the regional preference of patients, with two in-center sessions and two virtual sessions to assess the desired outcome.

Level of evidence: C; level of agreement: I - 93.83%, II - 5.56%, IV - 0.62%.

The 2023 Taiwan Myocardial Infarction Society/Taiwan Society of Cardiology/Taiwan Academy of Cardiovascular and Pulmonary Rehabilitation (TAMIS/TSOC/TACVPR) consensus statement recommends heart-friendly diets for patients with CVD, particularly after MI. These diets include low-fat, Mediterranean, DASH (Dietary Approaches to Stop Hypertension), or plant-based options, emphasizing reduced sugar, salt, and unhealthy fats, while also considering traditions, culture, religion, economics, and individual metabolic goals [37].

A nationwide cross-sectional electronic survey conducted in 2013 and 2015 evaluated dietary interventions in CR across Danish hospitals and municipalities. Results indicated disparities: 72% of municipalities offered dietary interventions in 2015, compared to 94% of hospitals (p=0.007). Screening for dietary interventions increased from 26% to 38% in hospitals and from 26% to 29% in municipalities, highlighting that implementing guidelines in clinical practice requires time and effort [38]. A study evaluated intensive CR (ICR) and standard CR (SCR) programs among high-risk CVD patients in real-world practice; it reported that ICR, which included a plant-based diet, stress management, and social support, achieved 96% adherence versus 68% for SCR. Only ICR led to reductions in body weight (3.4%), low-density lipoprotein cholesterol (11.3%), other atherogenic lipids, glycated hemoglobin, and systolic BP. After 12.6 months, major adverse cardiac events were less frequent in the ICR group (11% vs. 17%), especially HF hospitalizations (2% vs. 8%). The study concluded that a comprehensive ICR program, incorporating a plant-based diet and psychosocial management, is feasible and effective for high-risk CVD patients [39].

Another study evaluated a dietary education program for CR patients, including two nutrition and CV prevention seminars, a dietary habits questionnaire, and BMI, fasting glucose, and plasma lipids measurements at baseline and follow-up. The intervention group received a mid-term evaluation of nutrient intake and personalized educational reinforcement from a dietitian. This group showed significant reductions in daily caloric intake, weight, and BMI compared to the control group, suggesting the effectiveness of individual nutritional counseling in reducing CV risk factors [40]. Individuals with CVD often experience higher frailty levels, but CR can improve these levels [41]. A secondary analysis found that each 1% improvement in the frailty index from baseline to follow-up increased the likelihood of achieving CR goals by 2.7% (OR=1.027, 95% CI: 1.005, 1.048, p=0.014) [42]. However, studies utilizing HCR to assess improvements in frailty are still necessary.

An HCR trial (HYCARET) evaluated a 12-week hybrid exercise-based CR program in patients with CAD, assessing grip strength (handgrip), leg strength (chair stand test), and FC (6MWT) before and after the program. Results reported significant improvements in all measures, indicating grip strength, leg strength, and FC. These findings suggest that a hybrid exercise-based CR enhances muscle strength and FC [3]. Similarly, an HCR program integrating facility- and home-based exercises via a smartphone app showed significant 12-week improvements (p<0.05) in peak oxygen uptake, exercise capacity, and body fat percentage [43].

Statement: A hybrid program should include guidance recommending personalized exercise prescriptions to improve FC month by month, starting with two initial in-center sessions, followed by weekly individual and group virtual sessions.

Level of evidence: B; level of agreement: I - 89.51%, II - 8.64%, IV - 1.85%.

The 2023 TAMIS/TSOC/TACVPR consensus recommends low-intensity aerobic exercise in post-acute MI rehabilitation, with two to four sessions daily. Exercise should target a heart rate of 20 beats/minute above resting, up to 120 beats/minute, or a Borg Rating of Perceived Exertion (RPE) scale score of 13. Initial training includes three to five walking sessions using an ergometer or treadmill. Phase 2 CR for post-acute MI outpatients should include aerobic exercise three to five days/week, starting at 40% of exercise capacity and progressing to 80%. Intensity is monitored using heart rate, oxygen reserve, or the Borg scale (12-16 for patients on beta-blockers or with atrial fibrillation). Duration progresses from 20 to 60 minutes daily, with activities such as treadmill walking, cycling, and ergometer exercises [37].

Resistance exercise post-acute MI begins with two non-consecutive days/week, progressing to three. Intensity starts at 30%-40% of one-repetition maximum for the upper body and 50%-60% for the lower body, increasing as tolerated. The programs include one set initially, progressing to three sets of 10-15 repetitions for 8 to 10 exercises targeting major muscle groups. Modes include body weight, dumbbells, wrist weights, elastic bands, calisthenics, pulley or free weights, or weight machines. Phase 3 maintenance training is recommended at least three times a week for more than 30 minutes/session. Follow-up exercise testing is done to monitor progress, adjust prescriptions, and detect physiological changes [34].

Real-world evidence shows that using the Lyfe application improved physical activity among patients with CAD or ACS [35]. A multicenter, assessor-blinded RCT with 312 patients across the heart failure with preserved ejection fraction (HFpEF) spectrum evaluated the PRIORITY (Personalized Remotely Guided Preventive Exercise Therapy for a Healthy Heart) intervention. Participants were randomized 1:1 to a personalized, remotely guided preventive exercise prescription. The PRIORITY group completed 18 supervised, center-based exercise sessions over one year, complemented by a remotely guided home-based program. Outcomes were measured at baseline, four months, and at one and two years. The primary outcome was peak oxygen uptake at one year; secondary outcomes included physical activity, additional fitness parameters, CV health, echocardiographic measures, health-related QoL (HRQoL), and cost [44].

CR Team

Statement: A multidisciplinary team of experienced and credentialed cardiopulmonary therapists, clinical nutritionists, paramedic/cardiac nurses, psychosocial counselors, and disease-specific educators can support busy cardiologists in delivering CR components for desired patient outcomes.

Level of evidence: C; level of agreement: I - 100%.

The delivery of CR services typically involves a team comprising a cardiologist/physician co-coordinator, nurse specialist, physiotherapist, dietitian, occupational therapist, pharmacist, psychologist, smoking cessation counselor, social worker, vocational counselor, and administrative staff [45,46], offering patients support to manage lifestyle, behavior, and CV risk factors [47]. The HCR teams may also include a medical director, program director, exercise physiologists, nurses, researchers, preventive cardiologists, engineers, compliance/legal staff, and frontline clinicians [5]. Quality improvement programs like Cardiac Rehabilitation Phase II (CRP2) involve cardiologists, exercise physiologists, physiotherapists, nurses, pharmacists, occupational therapists, dietitians, and exercise trainers. This team composition is recommended in the 2023 TAMIS/TSOC/TACVPR consensus statement for patients with acute MI [17,35], as well as in Japanese Circulation Society (JCS) Joint Working Group guidelines for rehabilitation in patients with CVD [48].

The training project, titled “Multidisciplinarity in cardiac rehabilitation and secondary prevention: from the evaluation to the therapeutic education,” emphasizes comprehensive care for patients with ischemic heart disease. Cardiologists, nurses, dietitians, physiotherapists, and psychologists work together to promote lifestyle changes and reintegrate patients into daily lives and ensure coordinated care at every stage of assessment, intervention, and evaluation. The approach delivers patient-centered care encompassing therapeutic education and lifestyle management [49].

Outcome

Statement: Outcomes should be assessed based on improvements in bio-vitals and lab-vitals. The FC improvements should be measured by step count or the 6MWT test, and QoL should be evaluated using the QoL questionnaire.

Level of evidence: A; level of agreement: I - 98.15%, II - 1.23%, III - 0.62%.

A meta-analysis indicated that HCR resulted in greater improvement in peak oxygen uptake (9.72 mL/kg per minute; 95% CI: 5.12, 14.33) but no significant improvement in HRQoL (standardized mean difference: 0.67; 95% CI: -0.20, 1.54). Few studies reported similar improvements in FC (0.0 mL/kg per minute; 95% CI: -1.93, 1.92) and HRQoL (four studies; standardized mean difference: 0.11; 95% CI: -0.12, 0.34) when comparing HCR to center-based CR [6].

A prospective, two-arm, nonrandomized study compared traditional CR (which included supervised exercise sessions, health education classes, and a resource manual) with HCR (a blend of supervised and unsupervised exercise, independent home-based exercise, and follow-up phone calls). Both models showed significantly improved HRQoL, physical activity, diet, BP, cholesterol, waist circumference, and depressive symptoms (p<0.001). Both models were equally effective in improving cardiac health and behaviors [50].

Another study compared in-person, hybrid, and virtual CR using the six-minute walk test. All groups showed similar improvements in the 6MWT, BP control, and anxiety. However, virtual CR patients showed less improvement in depressive symptoms. Hybrid and virtual CR were comparable to in-person CR, supporting broader accessibility [34]. A study of a physical therapist-driven hybrid model for CR reported clinically meaningful improvements in 94.3% of patients for the 6MWT and 91.4% for the one-minute sit-to-stand test, demonstrating adherence, safety, and effectiveness [51]. A phase II CR program after coronary artery bypass grafting showed significant improvements in all HRQoL domains immediately after the program and at follow-up, indicating short-term and long-term enhancements in the physical, mental, and emotional aspects of patients' QoL [52].

**Figure 2:**
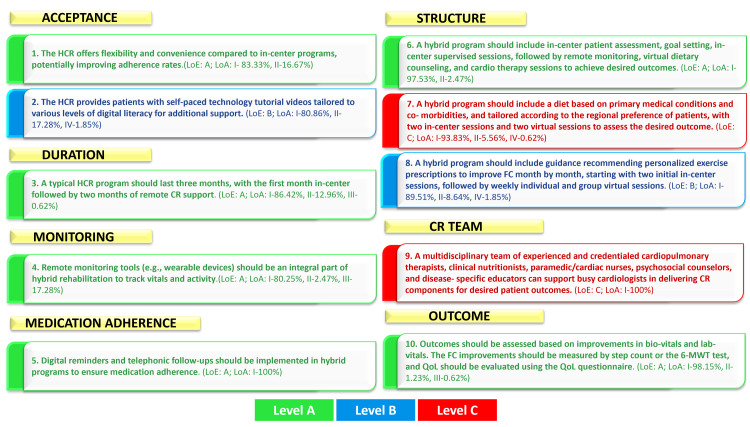
Summary of evidence supporting 10 statements from experts CR, cardiac rehabilitation; FC, functional capacity; LoA, level of agreement; HCR, hybrid cardiac rehabilitation; LoE, level of evidence (Level A, Level B, Level C); QoL, quality of life Image credits: Created by the authors using Microsoft PowerPoint (Microsoft Corporation, Redmond, USA)

A summary of 10 statements with the level of evidence and agreement is depicted in Figure 2. Also, the evolution of HCR is presented in Table 3.

**Table 3:** Key studies informing the development of consensus statements on hybrid cardiac rehabilitation BP, blood pressure; BMI, body mass index; CR, cardiac rehabilitation; CI, confidence interval; FBCR, facility-based cardiac rehabilitation; HRQoL, health-related quality of life; HBCR, home-based cardiac rehabilitation; CBCR, center-based cardiac rehabilitation; HCR, hybrid cardiac rehabilitation; RCT, randomized controlled trial; 1STS, one-minute sit-to-stand test; 6MWT, six-minute walk test.

Author, year	Study design	Interventions	Study outcomes
Keteyian et al., 2024 [13]	RCT	Patients attended 1 to 12 in-facility sessions, with the remaining completed virtually via two-way audiovisual technology.	HCR, which includes virtually supervised exercise, showed similar patient attendance and improvements in exercise capacity and health status as FBCR, making it a suitable alternative.
Naessens et al., 2024 [43]	Comparative study	Blend of supervised and home-based exercise sessions.	The HCR group as well as the CBCR group showed statistically significant (p<0.05) improvements in VO_2_ peak (mean +3.9 ml O_2_/kg/min vs. +3.2 ml O_2_/kg/min, respectively), peak load (mean +37.7W vs. +24.9W, respectively) and fat percentage (mean -2.14% vs. -1.17%, respectively) over the 12-week rehabilitation period.
Potdar et al., 2024 [35]	Prospective, single-center, RCT	Lyfe application.	The Lyfe group showed significantly greater improvements across all Dartmouth scale domains and had higher adherence to exercise (98% vs. 47.4%), diet (98% vs. 50%), and medication (92.5% rarely missed doses).
Amedro et al., 2024 [20]	Multicenter, RCT	12-week HCR program including a combined center- and home-based training design.	There was a significant positive change in the HRQoL total score (mean difference 3.8; 95% CI: 0.2, 7.3; p=0.038; effect size 0.34), BMI (mean difference -0.7 kg/m^2^; 95% CI: -1.3, -0.1; p=0.022; effect size 0.41), level of physical activity (mean difference 2.5; 95% CI: 0.1, 5; p=0.044; effect size 0.39), and disease knowledge (mean difference 2.7; 95% CI: 0.8, 4.6; p=0.007; effect size 0.51).
Minchin and Landers, 2024 [51]	Prospective single-group, time-series design	Physical therapist-driven hybrid model of the exercise component of CR that uses a novel intensity-recovery progression protocol and cardiac testing template.	Improvement beyond the minimal clinically important difference was 94.3% in the 6MWT and 91.4% in the 1STS.
Seron et al., 2024 [21]	Pragmatic, multicenter, parallel arm, open-label RCT	Participants received 10 center-based supervised exercise sessions plus counseling in 4 to 6 weeks, and then were supported at home via telephone calls and text messages through weeks 8 to 12.	In the intention-to-treat analysis, the hybrid CR group had 3.8% fewer cardiovascular events than standard CR, with a relative risk of 0.59. Per-protocol analysis at all adherence levels showed confidence intervals crossing the noninferiority margin.
Ganeshan et al., 2022 [34]	Comparative study	CR was delivered to patients in the hybrid and virtual cohorts using synchronous video exercise and/or asynchronous telephone visits.	Hybrid and virtual patients experienced similar improvements in BP control and anxiety. Virtual patients experienced less improvement in depression symptoms.
Gabelhouse et al., 2018 [50]	Prospective, two-arm, nonrandomized study	The hybrid model involved a blend of supervised and unsupervised, independent home-based exercise, and follow-up phone calls.	Significant improvements were observed for both models over time in HRQoL, physical activity, and diet (p<0.001). Significant reductions were observed in smoking (p=0.043), systolic BP, total cholesterol, low-density lipoprotein, waist circumference, and depressive symptoms (p<0.001).
Korzeniowska-Kubacka et al., 2014 [14]	Pilot study	The first 10 trainings were performed in a hospital, and the remaining 20 trainings were tele-monitored walking training at home (hybrid model).	Only workload and duration improved significantly. The home-based tele-monitored program supported patient adherence.

Discussion

The CR is often underutilized due to multiple barriers. Healthcare system factors, such as limited program availability, inadequate referral strategies, gaps in provider knowledge, and poor communication, restrict patient access. Social determinants of health, including economic status, education, employment, living conditions, food and housing insecurity, and insufficient insurance coverage, further influence CV outcomes and may limit patient engagement with CR programs [4,53].

To overcome these barriers, HCR has emerged as a flexible alternative. It combines home- and center-based sessions, which improves patients’ adherence to CR, enhances QoL, and thus reduces CV risk factors. However, there is no expert opinion or consensus from India on HCR. To address this gap, this study was conducted to review the existing literature and develop recommendations to guide Indian physicians in utilizing HCR to improve outcomes in patients with CVD.

The consensus statement highlights that HCR offers flexibility and convenience, thereby improving adherence rates. The program includes a combination of home- and center-based sessions, with one month at the center followed by two months of remote CR support. Key components of HCR include wearable devices for remote monitoring, digital reminders, and telephonic follow-ups to ensure medication adherence. The program incorporates in-center patient assessments, goal setting, supervision sessions, remote monitoring, virtual dietary counseling, and cardio therapy sessions aligned with the prescribed diet. It should also include personalized exercise prescriptions to improve FC month by month, with the involvement of a multidisciplinary team. Outcomes are assessed through bio-vitals, lab vitals, FC improvement, and QoL using a questionnaire.

The strength of this consensus document lies in the comprehensive review of the existing literature and expert opinions regarding the implementation of HCR. However, a lack of clinical data specific to India limits the validation of these recommendations within the Indian context. Establishing clinical evidence specific to India is crucial for the effective implementation of HCR.

Key take-home messages are as follows: (a) The HCR, combining home- and center-based care, offers flexible support that improves patient adherence, QoL, and CV outcomes. (b) A multidisciplinary team, including therapists, nutritionists, nurses, counselors, and educators, is essential to provide comprehensive, personalized care alongside cardiologists. (c) Patient progress should be tracked with vitals, lab tests, functional capacity, and QoL surveys. (d) Exercise and diet plans must be personalized to health needs and cultural preferences, delivered via both in-person and virtual sessions.

## Conclusions

The underutilization of CR has led to a new innovative approach that combines both home- and center-based sessions, known as HCR. This article presents the first Indian consensus on HCR, providing an unbiased summary of the existing data. The consensus strongly recommends the use of HCR in the management of CVD risk factors, which can ultimately improve patient outcomes and QoL.
